# Supply-side interventions to improve health: Findings from the Salud Mesoamérica Initiative

**DOI:** 10.1371/journal.pone.0195292

**Published:** 2018-04-16

**Authors:** Ali H. Mokdad, Erin B. Palmisano, Paola Zúñiga-Brenes, Diego Ríos-Zertuche, Casey K. Johanns, Alexandra Schaefer, Sima S. Desai, Annie Haakenstad, Marielle C. Gagnier, Claire R. McNellan, Danny V. Colombara, Sonia López Romero, Leolin Castillo, Benito Salvatierra, Bernardo Hernandez, Miguel Betancourt-Cravioto, Ricardo Mujica-Rosales, Ferdinando Regalia, Roberto Tapia-Conyer, Emma Iriarte

**Affiliations:** 1 Institute for Health Metrics and Evaluation, Seattle, Washington, United States of America; 2 Salud Mesoamérica Initiative / Inter-American Development Bank, Edificio Tower Financial Center (Towerbank), Panamá, Panamá; 3 UNIMER, San Francisco, San Salvador, El Salvador; 4 University of Belize, Belmopan, Belize; 5 El Colegio de la Frontera Sur, Barrio Maria Auxiliadora, San Cristóbal de las Casas, Chiapas, MÉXICO; 6 Fundación Carlos Slim, Delegación Miguel Hidalgo, México, Distrito Federal; 7 Inter-American Development Bank, NW, Washington, DC, United States of America; Western Oregon University, UNITED STATES

## Abstract

**Background:**

Results-based aid (RBA) is increasingly used to incentivize action in health. In Mesoamerica, the region consisting of southern Mexico and Central America, the RBA project known as the Salud Mesoamérica Initiative (SMI) was designed to target disparities in maternal and child health, focusing on the poorest 20% of the population across the region.

**Methods and findings:**

Data were first collected in 365 intervention health facilities to establish a baseline of indicators. For the first follow-up measure, 18 to 24 months later, 368 facilities were evaluated in these same areas. At both stages, we measured a near-identical set of supply-side performance indicators in line with country-specific priorities in maternal and child health. All countries showed progress in performance indicators, although with different levels. El Salvador, Honduras, Nicaragua, and Panama reached their 18-month targets, while the State of Chiapas in Mexico, Guatemala, and Belize did not. A second follow-up measurement in Chiapas and Guatemala showed continued progress, as they achieved previously missed targets nine to 12 months later, after implementing a performance improvement plan.

**Conclusions:**

Our findings show an initial success in the supply-side indicators of SMI. Our data suggest that the RBA approach can be a motivator to improve availability of drugs and services in poor areas. Moreover, our innovative monitoring and evaluation framework will allow health officials with limited resources to identify and target areas of greatest need.

## Introduction

Mesoamerica, the region consisting of southern Mexico and Central America, has made significant progress in population health over the past decade, though substantial inequalities in health outcomes, access, and quality of medical care remain between and within countries [1–5]. In these areas, vulnerable groups such as poor, indigenous, and rural populations have considerably worse health outcomes than national or regional averages [6–16].

The Salud Mesoamérica Initiative (SMI) was established to address the health issues faced by the poorest quintile of the population in El Salvador, Guatemala, Honduras, Nicaragua, Belize, Costa Rica, Panama, and the State of Chiapas, Mexico. Administered by the Inter-American Development Bank (IDB), SMI uses a results-based aid (RBA) approach to improve maternal and child health across the region. The initiative is a public-private partnership including the Bill & Melinda Gates Foundation, the Carlos Slim Foundation, Spain’s Cooperation Agency for International Development, and ministries of health in each of the eight participating countries.

RBA and results-based financing (RBF) are financial assistance models involving contractual arrangements between a principal and an agent and involve the transfer of funds in exchange for delivery of pre-specified performance targets or results [17–20]. The approaches differ by the defined source of funding and contractual partners involved. In typical RBF for health, contracts are executed between a government and an implementing partner at the sub-national level and financial incentives are often applied at the facility level. The RBA model includes a financial assistance contract between a donor and a national government, where either a portion of or all donor funds are disbursed based upon performance or predetermined targets [17–21].

Very few RBA models for health exist and due to its novelty, success has not yet been confirmed. The evaluation of the Gavi Immunization Services Support (ISS) grants in Cambodia, Laos, Tanzania, Zambia, Democratic Republic of the Congo, and Guinea cites an association between ISS funding and immunization coverage rates but provides limited data to assess the effectiveness of the RBA component of the program [22]. In this case, it is not possible to ascertain the direct effect of the RBA given the lack of specific controls in the evaluation design. A literature review of major results-based financial assistance schemes suggest that they generally appear to deliver results, but that there is extremely weak evidence to attribute results specifically to the RBA/RBF component [17].

Several studies have evaluated RBF programs in the health sector and have provided mixed results [23–34]. Systematic reviews cite some RBF evaluations which present no effect or suggest potential for negative outcomes, such as perverse incentives, gaming, and technical difficulties [17,23]. At the same time, many evaluations suggest promising results, implying that RBF can be an important tool for achieving health targets as well as health sector reform more broadly, if designed carefully [23–25].

Key factors have been identified for achieving success in results-based programs, including political commitment and a participatory approach, clearly defined rules, indicators, and targets, strong verification systems, and robust measuring and evaluation [23,24,34]. Improvements in health service utilization and perceived quality of care indicators were observed from RBF programs in Burundi, the Democratic Republic of the Congo, Tanzania, and Zambia [29]. Engagement with local and national health management from the onset of a program is cited as a crucial component for RBF; programs that did not deploy such an approach were less successful in strengthening health systems [29]. Furthermore, the evaluation of an RBF program to increase the use and quality of child and maternal care services in Rwanda found that within 23 months, the program led to increased use and quality of health services [30].

The design of SMI as an RBA is different from previous studies. Participating countries receive 50% of the cost of program intervention from funders and contribute the remaining 50% themselves. At the end of each operation, pre-defined performance indicators are measured independently, and if 80% of these indicators are met, the country is awarded half of its contribution share back. Funds awarded to recipient countries for meeting targets can be spent on other health programs at the country’s discretion. SMI’s design enables IDB to dispense these pre-agreed funds to countries based on their compliance with country-specific health targets in selected geographic areas. SMI uses this RBA approach and delivers integrated evidenced-based supply- and demand-side interventions to improve maternal and child health for the poorest quintile of the population across Mesoamerica.

In this manuscript, we describe the improvement from the baseline to the first follow-up supply-side measurements of SMI.

## Methods

The SMI methodology was previously published [35,36]. Briefly, the IDB worked with countries to select the poorest regions for the intervention. The selection was based on national poverty measures, roughly equivalent to the bottom 20% of the income or wealth distribution. A scale was created to order health jurisdictions and municipalities according to the country-defined poverty metrics and to select municipalities eligible for intervention from the poorest quintile. Selection from these groupings of municipalities also considered additional criteria, such as the presence of gaps in health service coverage, the existence of ongoing health reforms, and the presence of complementary financing by other donors and the government. In each country, SMI is implementing two to three consecutive projects. Each project includes a set of performance (payment) indicators and targets that were set with governments, in line with country-specific priorities in maternal and child health. Key indicators include coverage of contraceptives, antenatal and postnatal care for women and newborns, in-facility delivery and skilled birth attendance, management of maternal and neonatal complications, complete vaccination coverage for age, prevalence of anemia in children, and quality of care for antenatal, delivery, postnatal, and child health care visits. As part of establishing the baseline performance for SMI indicators, surveys were conducted in households and health facilities in each country.

Written informed consent was obtained from all participants. The study received approval from the institutional review board (IRB) from the University of Washington, partnering data collection agencies, and the Ministry of Health in each country.

Interventions included implementing the Essential Obstetric and Neonatal Care (EONC) strategy, strengthening referral networks, improving the supply chain, encouraging the cultural adaptation of services for indigenous populations, supporting new service delivery platforms and community platforms, and the design and approval of updated country norms and protocols, among other activities. SMI design calls for follow-up measurements at the close of each of three intervention periods in each country to capture the impact of the interventions. Depending on whether targets for improvement are met at each of these critical junctures, countries will be reimbursed with funds corresponding to half of the counterpart investment that they had to put forward up front, to be used freely within the health sector.

SMI dispensed about $23 million for the first operation, with similar matching funds coming from the participating countries (S1 Table). A tranche equal to half of the government contribution was awarded if a country met the 18-month targets.

Indicator targets were set through negotiation with the countries’ health officials and IDB based on literature reviews of intervention effectiveness from previous country-level studies, trend analysis using data from the Global Burden of Disease 2010 study [2, 37–41], expert advice, and a cost-benefit model developed by IDB. Several health facility indicators were used by SMI and measured at the baseline and 18-month junctures (S2 Table). Performance indicators for payment varied in their requirements by country (S3 Table shows the most comparable ones). For example, the indicator related to the supply of antenatal and postnatal care, “health facilities with permanent availability of inputs and equipment necessary for prenatal and postpartum care” had five components in El Salvador compared to 29 in Belize due to negotiation and agreement between each country and IDB. In order for a country to “pass,” 80% of facilities must meet the indicator targets. For a facility to be declared as having met an indicator, every single component of that indicator must be met. For the 18-month measurement, countries committed to a predetermined target for each supply-side performance indicator. Targets were set specific to each country and were quantified as a percentage of facilities complying with all indicator components.

For each round, we selected a random sample of the facilities to be representative. All facilities were eligible for inclusion in each round. We conducted a third round of measurement for Chiapas and Guatemala, for the performance indicators only, after both implemented a performance improvement plan, given that they failed to achieve their targets at 18 months but they showed improvement. We only measured the performance indicators during the repeat to reduce the cost of the measurement. Though Belize also did not meet 18-month targets, a third round measurement was not conducted due to the limited SMI investment funds in Belize, as detailed in S1 Table.

The health facility surveys at baseline and 18 months collected data on facility conditions, service provision and utilization, and quality of care. The survey involved three main components: an interview questionnaire, an observation checklist, and medical record reviews (MRRs). Health facilities were grouped according to three EONC levels—ambulatory, basic, and complete—as provided by SMI. Mainly, ambulatory level facilities have the capacity to provide outpatient care only; basic-level facilities are able to attend uncomplicated vaginal deliveries and provide immediate emergency obstetric and neonatal care; and complete-level facilities have a surgery room and the capacity to attend most obstetric and neonatal complications (not including intensive care). Different health facility indicator criteria were assessed depending on the EONC classification level. In the interview questionnaire, the facility director, manager, or other person in charge of the health facility was interviewed to capture information on general facility characteristics, infrastructure, human resource composition and trainings, supply logistics, infection control, child health care provision, vaccine availability, family planning service provision, availability of contraceptives, and antenatal, delivery, and postpartum care. Once these were completed, surveyors used an observation checklist to record direct observations of the availability and functionality, as applicable, of essential equipment and supplies for maternal and child health care, including pharmaceuticals. Surveyors also reviewed administrative records of pharmaceutical stocks in this module, capturing drug and contraceptive stock-outs occurring in the three months prior to the date of the survey.

We used MRRs to capture retrospective data on record-keeping and treatment practices of surveyed facilities. MRRs were selected at random from each facility using the records of encounters and applying systematic sampling to cover the entire study period. The MRRs covered various medical complications that mothers and infants experienced during delivery and how each sampled case was treated at a given health facility. The MRRs also captured the facilities’ medical practices before, during, and after uncomplicated births. Depending on the country, other MRRs on diarrhea, pneumonia, low birth weight, child registration and growth charting, deworming, and family planning services offered were also implemented. Indicators from MRRs were collected for monitoring purposes during the 18-month measurement but will be included as performance- or payment-based indicators in the subsequent evaluation.

The SMI surveys were conducted using a computer-assisted personal interview (CAPI), and incoming data were continuously monitored by the Institute for Health Metrics and Evaluation (IHME). We conducted training sessions and pilots in each country before implementation. We also conducted supervisory visits during health facility data collection. We used Stata 13.1 for the analyses and to account for the complex study design. Baseline surveys were conducted from March 1, 2011, to August 31, 2013, while the 18-month follow-up surveys were conducted between January 20, 2014, and October 24, 2014. Dates of the surveys were designed to ensure an 18-month period between the baseline and the follow-up for each country. Performance improvement plan measurements (PIPM) in Chiapas and Guatemala were conducted between May 11, 2015, and June 25, 2015. Additional detail on SMI methodology and implementation is available elsewhere [35].

## Results

In total, 365 health facilities were interviewed for the baseline measurement, and 6,332 medical records were reviewed, compared to 368 and 11,143, respectively, for the 18-month follow-up (Table 1). Moreover, 30 facilities were measured as part of the performance improvement plan nine to 12 months after the 18-month follow-up in Guatemala and Chiapas.

**Table 1:** Health facility survey sample description.

Country	Baseline		18-month follow-up	
	Facilities	Medical records	Facilities	Medical records
Belize	39	792	38	1,190
El Salvador	65	n/a	60	1,591
Guatemala	64	1,175	60	2,299
Honduras	59	1,293	60	1,517
Chiapas (Mex.)	60	1,724	60	1,985
Nicaragua	40	850	60	1,698
Panama	38	498	30	863
**Total**	**365**	**6,332**	**368**	**11,143**

Table 2 shows the progress in selected payment indicators from baseline to 18 months and related indicator targets. All countries showed progress in the indicators, although with different levels. El Salvador, Honduras, Nicaragua, and Panama reached their 18-month targets, while Chiapas, Guatemala, and Belize did not. The third measurement showed that both Guatemala and Chiapas reached their 18-month targets nine to 12 months later.

**Table 2:** Percentage of health facilities meeting selected health facility performance indicators and 18-month targets, by country.

		Health facilities with cold chain managed according to standards % (95% confidence interval (CI))	Health facilities with permanent availability of supplies and equipment necessary for pediatric, vaccination, and nutrition care* % (95% CI)	Health facilities with permanent availability of supplies and equipment necessary for prenatal and postpartum care % (95% CI)	Health facilities that have the necessary supplies and equipment for providing emergency obstetric and neonatal care according to the norms % (95% CI)	Health facilities with permanent availability of modern family planning supplies (oral, injectable, barrier, IUD) according to the norms** % (95% CI)
Belize	Baseline	n/a	0% (0–9·5%)	2·9% (0–14·9%)	0% (0–60·2%)	73·7% (48·8–90·9%)
	Follow-up	n/a	0% (0–9·5%)	17·2% (5·8–35·8%)	0% (0–60·2%)	90% (68·3–98·8%)
	Target	n/a	85%	85%	75%	85%
El Salvador	Baseline	n/a	36·2% (24–49·9%)	48.3% (35–61·8%)	n/a	19% (9·9–31·4%)
	Follow-up	n/a	92·2% (81·1–97·8%)	98% (89·6–100%)	n/a	92·2% (81·1–97·8%)
	Target	n/a	75·3%	88.3%	n/a	84·4%
Guatemala	Baseline	n/a	3·2% (0·4–11·0%)	14·1% (6·6–25·0%)	0% (0–19·5%)	59·7% (46·4–71·9%)
	Follow-up	n/a	3·6% (0·4–12·5%)	10·7% (4·0–21·9%)	8·3% (0.2–38·5%)	65·5% (51·4–77·8%)
	Target	n/a	50%	50%	50%	70%
Honduras***	Baseline	n/a	0% (0·0–4·6%)	n/a	62·5% (24·5–91·5%)	86·4% (75·0–94·0%)
	Follow-up	n/a	51·1% (35·8–66·3%)	n/a	85·7% (42·1–99·6%)	93% (83.0–98·1%)
	Target	n/a	80%	n/a	80%	90%
Chiapas (Mex.)	Baseline	70·8% (48·9–87·4%)	3·6% (0·4–12·5%)	3·6% (0·4–12·5%)	0% (0–24·7%)	55·1% (40·2–69·3%)
	Follow-up	77·8% (57·7–91·4%)	13·6% (6·0–25%)	45·8% (32·7–59·2%)	14·3% (1·8–42·8%)	62·7% (49·1–75%)
	Target	80%	80%	80%	80%	80%
Nicaragua***	Baseline	28·6% (13·2–48·7%)	0% (0–9·5%)	10·8% (3·0–25·4%)	60% (14·7–94·7%)	59·5% (42·1–75·2%)
	Follow-up	88·9% (70·8–97·6%)	71·7% (57·7–83·2%)	76·8% (63·6–87·0%)	90·9% (58·7–99·7%)	87·5% (75·9–94·8%)
	Target	85%	85%	85%	85%	85%
Panama	Baseline	n/a	11.8% (1.5–36.4%)	17.6% (3.8–43.4%)	n/a	7·1% (0·2–33·9%)
	Follow-up	n/a	84.2% (60.4–96.6%)	100% (82.4–100%)	n/a	78·9% (54·4–93·9%)
	Target	n/a	80%	80%	n/a	80%

*The Honduras definition is health facilities with permanent availability of supplies and equipment for the treatment of pneumonia and diarrhea. This is only applicable for Honduras, as all other countries define this indicator more broadly as availability of supplies and equipment necessary for pediatric, vaccination, and nutrition care.

**Guatemala family planning indicator definition of target was reversed from percentage of facilities with stock-out to percentage of facilities without stock-out in order to show comparability across countries.

***Though Honduras and Nicaragua did not meet respective targets for the child care indicator, each country met 80% of performance indicators, achieving their overall 18-month target. Only some SMI performance indicators are presented in this table, considering indicators with comparability across countries. Additional country-specific performance indicators were captured during the 18-month assessment, which factored into whether or not countries met the 80% threshold.

**Fig 1:**
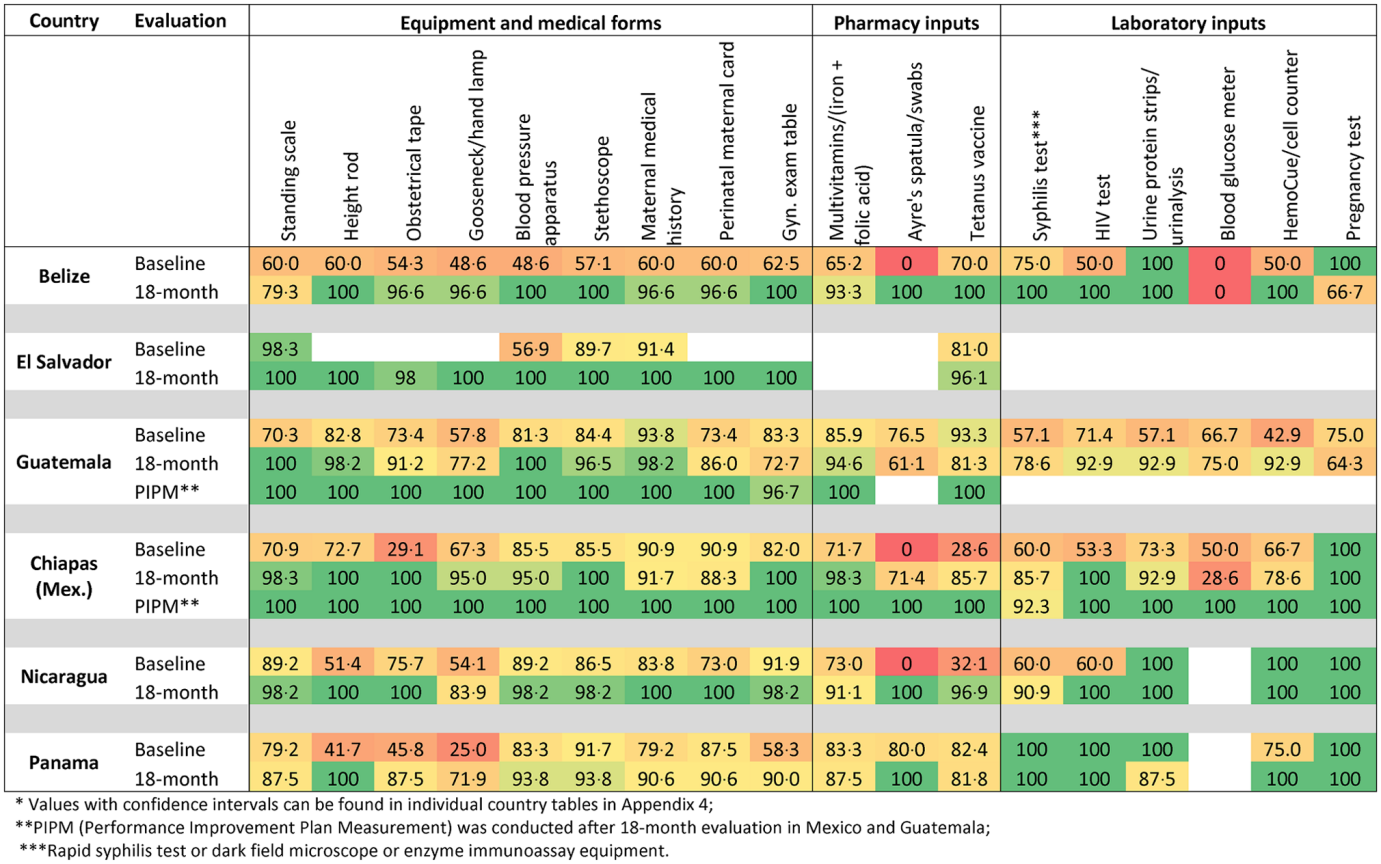
Percentage of facilities with selected equipment and supplies needed for the provision of basic antenatal and postnatal care services*.

All countries showed improvement in SMI indicators from the baseline to the 18-month measurements (S1 Appendix). In total, 448 individual components were measured for the 18-month performance indicators for the seven countries, and only 47 showed a decline, 72 showed no change, and 279 improved from the baseline (Table 3). Fig 1 shows a heat map of the improvement in selected components of the SMI indicator measuring the availability of equipment, pharmaceuticals, and lab inputs necessary for antenatal and postpartum care. Fig 1 details, by country and measurement round, the overall percentage of facilities that had specific components of the antenatal and postnatal care indicator. A clear improvement was observed in the availability of supplies, in all countries.

**Table 3:** Total number of components included in performance indicators measured through health facility surveys in the 18-month evaluation.

	Total number of payment indicators measured through surveys*	Total number of individual components included in payment indicators measured through surveys*,**	Percent of components improved from baseline	Percent of components decreased from baseline	Percent of components that neither improved nor decreased from the baseline	Percent of new components (not previously measured at the baseline)
Belize	8	105	56.2%	5.7%	25.7%	12.4%
El Salvador	7	32	59.4%	12.5%	15.6%	12.5%
Guatemala	6	76	48.7%	19.7%	7.9%	23.7%
Honduras	5	53	58.5%	5.7%	34.0%	1.9%
Chiapas (Mex.)	6	96	82.3%	15.6%	2.1%	0%
Nicaragua	5	43	74.4%	2.3%	16.3%	7.0%
Panama	8	45	51.1%	6.7%	17.8%	24.4%
Total	45	448	62.3%	10.5%	16.1%	11.2%

*The number of indicators and the number and type of components included in indicators varied by country based on negotiations between IDB and countries.

**Pharmacy supplies are represented considering availability only on the day of the survey for the purposes of this table; stock-out of various items in the previous three months were not considered as additional inputs for this table but are often included in performance indicator criteria.

**Fig 2:**
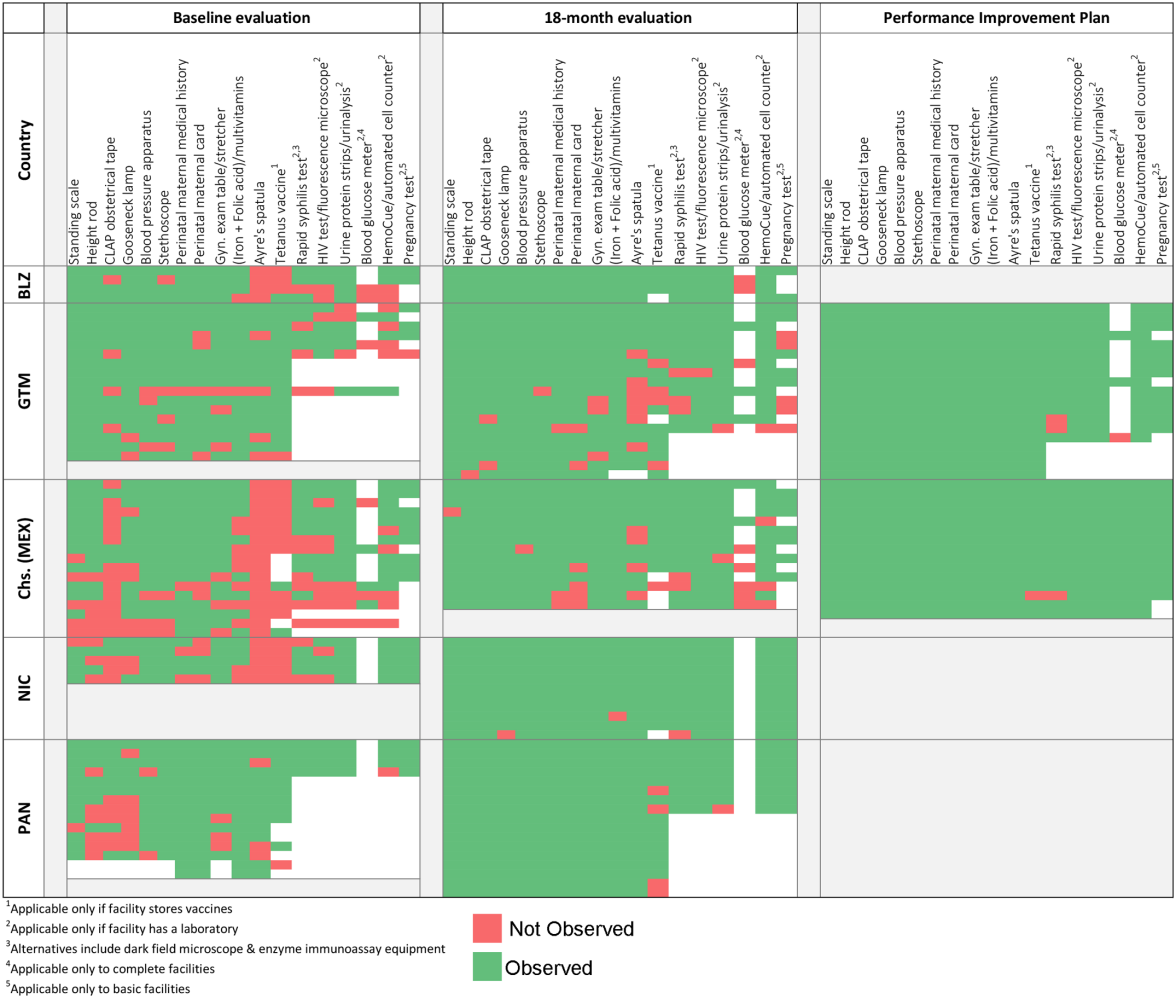
Basic and complete health facilities with selected equipment and supplies needed for the provision of basic antenatal and postnatal care service.

**Fig 3:**
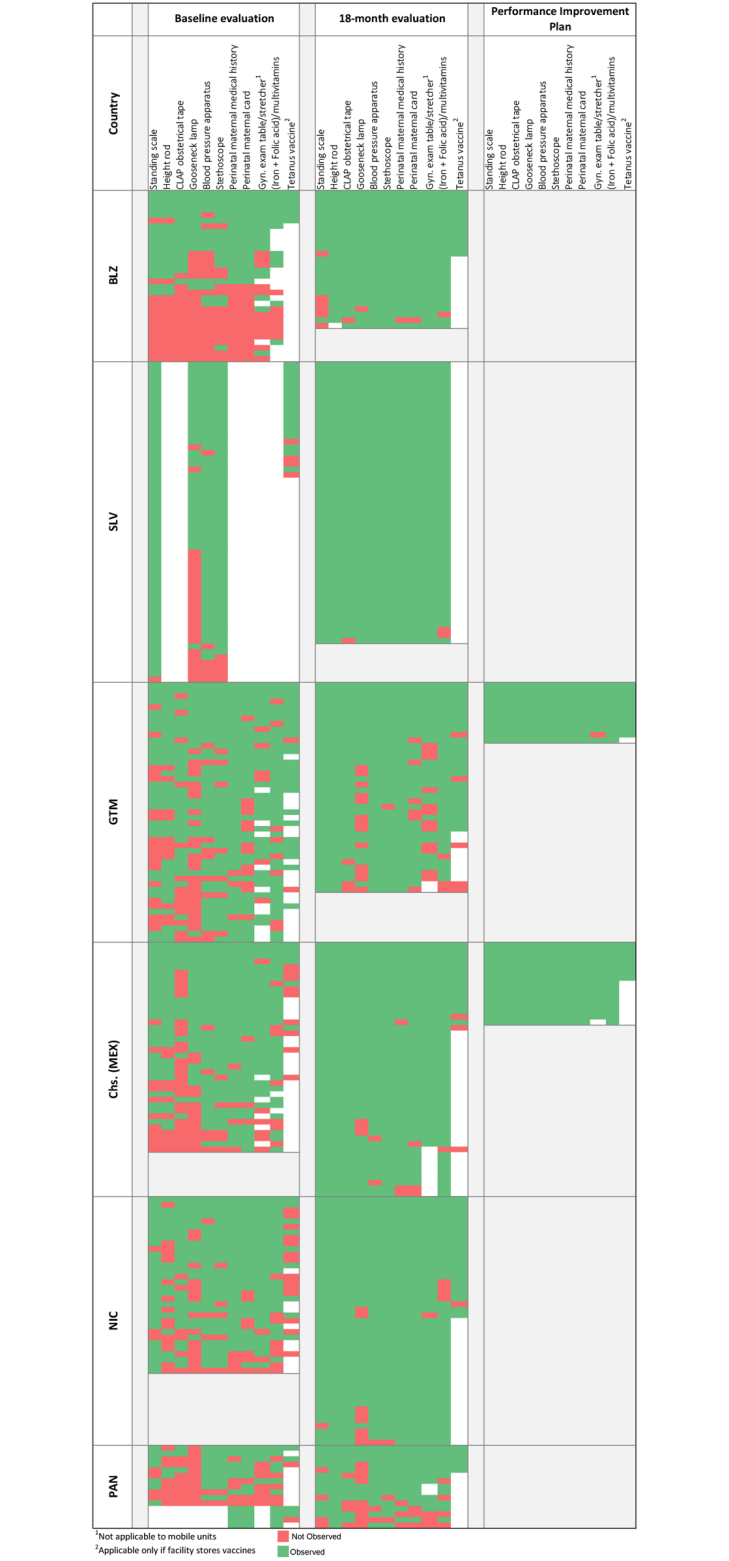
Ambulatory health facilities with selected equipment and supplies needed for the provision of basic antenatal and postnatal care service.

When we examined gains in equipment and supplies by health facilities, progress had been achieved in 18 months (Figs 2 and 3). Most facilities that did not meet the goal and qualify for disbursement of the performance tranche were missing only one or two components of complex indicators.

## Discussion

To our knowledge, this is the largest RBA project to improve supplies and services at health facilities in poor areas of Mesoamerica. Our data show improvement in the availability of supplies and medicines at health facilities in a short time. SMI data suggest that the RBA approach is a motivator to improve availability of drugs and services in poor areas. Our results are encouraging for public health as this improvement happened in poor and remote areas, indicating that lessons learned from SMI could be applied in other regions of these countries and globally.

SMI is a very ambitious project, and the targets set for each country were strict. For a facility to pass, it had to have all components of an indicator observed or achieved. The rule for disbursing the performance tranche was designed as a pass or fail, irrespective of the level of improvement in each facility, and was designed to offer a reward for extraordinary effort in a short time. Belize, Guatemala, and Chiapas did not pass the 18-month measurement, while Panama, Nicaragua, Honduras, and El Salvador did. The design of SMI ensures that the intervention continues in all countries, even though some countries did not receive a performance tranche at 18 months. However, improvement in supplies and equipment was observed in the three countries that did not meet their targets at the 18-month juncture. Our data show that most facilities in these countries did not pass because they were missing only one or two components of complex indicators. When both Guatemala and Chiapas were given an extra nine to 12 months to implement a performance improvement plan, they were able to meet all targets.

Although RBA/RBF is increasingly used to incentivize action in health, most results-based evaluations provide evidence for schemes that apply financial incentives at the health facility or health provider level. Less evidence is available on the impact of RBA approaches that provide incentives at the national level, such as the Gavi ISS program [22].

Our supply-side results add to the limited available evidence of the effect of RBA schemes that apply financial incentives at the national level. Our study shows an improvement in the availability of supplies and equipment at facilities in 18 months, though we are not yet able to show an increase in uptake of services by the population, since the SMI measurement calls for another household survey in 2017–2018. However, increasing availability of supplies and equipment has been associated with increases in uptake previously [42]. Subsequent rounds of evaluation of SMI will provide more robust evidence to this area of study. Continued rigorous evaluation is needed to assess successes and failures in RBA schemes where financial incentives are applied at the national level.

SMI raised the bar on the requirement of supplies and equipment in poor and remote areas. These demanding indicators should be maintained and used in future SMI follow-up measurements. For example, the presence of stethoscopes was a requirement, though many facilities that reported not owning a stethoscope were relying on facility staff’s personal stethoscopes. A strong argument could be made that stethoscopes should be available at the facilities since doctors or other facility staff may be absent on certain days (vacation or illness). Moreover, the improvement in such a strict and ambitious intervention in poor and remote areas is encouraging and suggests that such an approach could easily be adopted in other parts of each country. Showing decision-makers and health authorities that success can be achieved through proper strategies can ensure improvement in health elsewhere.

Our findings showed that countries had more success with certain components of an indicator. This was not surprising given the number of components and indicators we have in our evaluations. Our ability to provide each country with a detailed report on what are the missed targets and in what facilities will enable them to address these shortages. Moreover, our approach for evaluation could be used by countries to track their own progress in future years.

SMI provided a platform for action to improve health, giving all countries technical assistance to help remove bottlenecks, improve processes, analyze failures, and adjust strategies. Challenges and solutions for overcoming them should be documented, as should failures, to ensure that they are not repeated. After Chiapas and Guatemala did not meet targets and were given the external measurement results, they proved through the PIPM to be able to mobilize quickly in order to supply and equip facilities that were lacking. There is a need to include a qualitative component to this evaluation to complement the quantitative findings in order to better understand the drivers of success. Indeed, finding a way for people in remote areas to communicate their experience and provide the lessons learned is essential. Our findings call for creating a network of local health officials and residents to exchange ideas through the implementation of SMI.

Several factors have to be considered in SMI. First, as supplies increase in one area of the community (intervention areas), it is possible that the demand would increase from the community and neighboring ones, especially if they are not part of the intervention. Previous studies have shown that individuals may travel a longer distance to seek care at a better-equipped facility. Hence, these facilities could face a challenge in supplies and time going forward. Second, it is possible that the attention to comply with targets in the intervention areas has led to diverting resources to these areas at the expense of others, although we have no evidence that this occurred. Still, both of these scenarios have to be examined to ensure continued success.

Finally, a major concern for SMI is sustainability. Although the governments have agreed to provide funds to sustain the programs and intervention, it is possible that when donor investment and reimbursement end and the measurements stop things may change. A challenge for SMI is how to maintain these improvements once the funds from IDB are dispensed and IHME monitoring is complete. The sustainability of programs has been a major challenge in global health. However, we have already observed that governments have started their own monitoring of supplies and equipment. In fact, our interviewers in Chiapas and Guatemala documented how each component of country-specific indicators was numbered and labeled at some health facilities. Moreover, we hope that supply-side improvements will generate health-seeking behaviors and public and official awareness of the need to continue and maintain these achievements. Indeed, the population’s expectations will result in increased demands for these supplies and availability at their health facilities.

Our study has several limitations. First, we did not conduct a household survey with the health facility survey this measurement round, and thus were not able to examine the change in demand. However, this is a planned activity for the next round of the evaluation. Second, we did not conduct a qualitative assessment in all countries to explain our quantitative results and identify the drivers or obstacles for change. Indeed, our qualitative work in Chiapas showed that the RBA model created a culture of accountability, which was strengthened following the failure to meet targets during the first measurement round. With the improvement plan, accountability trickled down to the level of health workers, who developed the habit of routinely checking supplies and ensuring shortages were avoided [36]. Third, it is possible that we have some self-reporting bias in our survey. However, this does not affect most of our indicators, which are based on observation and MRRs. Finally, we did not examine indicators in control areas during this round of health facility surveys to see if the same improvements in supplies occurred outside our study area. However, subsequent rounds of measurement include data collection activities in both our comparison and study areas.

Our study showed that when local staff, central health, and federal officials set a priority and are given a monetary and a professional incentive, positive changes could occur in a short time and in a challenging environment. We found that this type of RBF is the right approach to improve health in Mesoamerica. Our findings have great implications on health funding for reducing the burden of diseases.

## Supporting information

S1 Table SMI first and second operation resources*. (DOCX)

S2 Table SMI 18-month performance indicators by country. (DOCX)

S3 Table SMI selected 18-month performance indicators and requirements by country. (DOCX)

S1 Appendix Improvements in supplies and equipment from baseline to 18-month follow-up, by country. (DOCX)
