# Supplementary material for: Supply-side interventions to improve health: Findings from the Salud Mesoamérica Initiative
Source: PLoS One. 2018 Apr 16;13(4):e0195292. doi: 10.1371/journal.pone.0195292 (PMC5901783; doi:10.1371/journal.pone.0195292)
Supplement: S1 Table — (DOCX) [file pone.0195292.s001.DOCX]

**S1 Table. SMI first and second operation resources***

|  | First Operation Resources (USD) | | | Second Operation Resources (USD) | | | Total Resources (USD) | | |
| --- | --- | --- | --- | --- | --- | --- | --- | --- | --- |
|  | Initiative funding | Government counterpart | Performance Tranche*** | Initiative funding | Government counterpart | Performance Tranche*** | Initiative funding | Government counterpart | Performance Tranches*** |
| Belize** | 500,000 | 500,000 | 250,000 | 250,000 | 250,000 | 125,000 | 750,000 | 750,000 | 375,000 |
| Costa Rica**** | 1,142,857 | 1,714,286 | 857,143 | 846,599 | 1,269,898 | 634,949 | 1,989,456 | 2,984,184 | 1,492,092 |
| El Salvador | 4,875,000 | 3,250,000 | 1,625,000 | 2,958,484 | 1,972,323 | 986,161 | 7,833,484 | 5,222,323 | 2,611,161 |
| Guatemala | 5,830,000 | 3,886,667 | 1,943,333 | 7,804,645 | 5,203,096 | 2,601,549 | 13,634,645 | 9,089,763 | 4,544,882 |
| Honduras | 4,000,000 | 4,000,000 | 2,000,000 | 3,500,000 | 3,500,000 | 1,750,000 | 7,500,000 | 7,500,000 | 3,750,000 |
| Chiapas (Mex.) | 2,585,906 | 3,878,857 | 1,939,428 | 2,585,904 | 3,878,857 | 1,939,429 | 5,171,810 | 7,757,714 | 3,878,857 |
| Nicaragua | 3,466,949 | 2,311,299 | 1,155,650 | 2,724,030 | 1,816,021 | 908,010 | 6,190,979 | 4,127,320 | 2,063,660 |
| Panama | 1,142,857 | 1,714,286 | 857,143 | 1,167,479 | 1,751,218 | 875,609 | 2,310,336 | 3,465,504 | 1,732,752 |
| Total | 23,543,569 | 21,255,395 | 10,627,697 | 21,837,141 | 19,641,413 | 9,820,707 | 45,380,710 | 40,896,808 | 20,448,404 |
| *Data taken from the Salud Mesoamérica website [43].  ** Given the limited available funds for the Belize operation, we did not conduct a repeat evaluation after the 18-month measurement as we did not want to spend limited resources when knowing that there would be a third measurement conducted in all countries in 2017-2018.  ***Performance Tranche is funded by SMI and is dependent on meeting predefined indicator targets.  ****The SMI operation in Costa Rica focuses on adolescent reproductive health with the majority of data collected through school surveys. For this reason, Costa Rica is not detailed in the results section of the paper for increases in supply and equipment at health facilities. | | | | | | | | | |

**Reference:**

43. Resources [Internet]. Iniciativa Salud Mesoamérica. Available from: http://www.sm2015.org/en/salud-mesoamerica-initiative/the-initiative/resources,6585.html
