# Supplementary material for: Supply-side interventions to improve health: Findings from the Salud Mesoamérica Initiative
Source: PLoS One. 2018 Apr 16;13(4):e0195292. doi: 10.1371/journal.pone.0195292 (PMC5901783; doi:10.1371/journal.pone.0195292)
Supplement: S2 Table — (DOCX) [file pone.0195292.s002.docx]

**S2 Table. SMI 18-month performance indicators by country**

| Indicator # | Performance indicator description | BLZ | SLV | GTM | HND | CH (MEX) | NIC | PAN |
| --- | --- | --- | --- | --- | --- | --- | --- | --- |
| 7000 | Health facilities with cold chain managed according to standards |  |  |  |  | X | X |  |
| 7001 | Health facilities with availability of a functioning refrigerator |  | X |  |  |  |  |  |
| 7010 | Health facilities with permanent availability of supplies and equipment necessary for pediatric, vaccination, and nutrition care | X | X | X |  | X | X | X |
| 7020 | Health facilities with permanent availability of supplies and equipment necessary for prenatal and postpartum care | X | X | X |  | X | X | X |
| 7030 | Health facilities that have the necessary supplies and equipment for providing emergency obstetric and neonatal care, according to the norms | X |  | X | X | X | X |  |
| 7035 | Hospitals that have the necessary supplies and equipment for providing emergency obstetric and neonatal care, according to the norms |  |  |  | X |  |  |  |
| 7040 | Health facilities with permanent availability of supplies and equipment necessary for delivery and newborn care |  |  |  |  | X |  | X |
| 7050 | Health facilities with permanent availability of modern family planning supplies (oral, injectable, barrier, IUD), according to the norms | X | X | X | X | X | X | X |
| 7060 | Health facilities with permanent availability of supplies and equipment for the treatment of pneumonia and diarrhea |  |  |  | X |  |  |  |
| 7070 | Health facilities with the availability of powdered micronutrients |  |  |  | X |  |  |  |
| 7160 | Municipal Health Districts that can access data and generate regular reports in immunization, maternal, newborn, and child care |  |  | X |  |  |  |  |
| 7192/7193 | Health facilities with availability of specified staff |  | X |  |  |  |  | X |
| 7410 | Health facilities that have implemented Quality of Care job aid tools for reproductive health | X |  |  |  |  |  |  |
| 7420 | Health facilities that have sexual and reproductive health (SRH) educational materials specifically targeted at adolescents | X |  |  |  |  |  |  |
| 7460 | Health facilities with a mechanism in place for carrying out patient satisfaction surveys | X |  |  |  |  |  |  |
| 7465 | Health facilities that can submit and receive data from the Belize Health Information System (BHIS) | X |  |  |  |  |  |  |
| 7710 | Health facilities that possess monthly AIN-C registries in which 80% of children under 24 months are registered with weight |  |  |  |  |  |  | X |
| 7730 | Health facilities with population living in communities with a plan for better community sanitation and water quality |  |  |  |  |  |  | X |
| 7740 | Health facilities with birthing plans for communities under their responsibility (which have been approved by the community) |  |  |  |  |  |  | X |
| 8610 | Children aged 0-23 months who received growth monitoring according to their age in their most recent visit |  |  | X |  |  |  |  |
