# Supplementary material for: Supply-side interventions to improve health: Findings from the Salud Mesoamérica Initiative
Source: PLoS One. 2018 Apr 16;13(4):e0195292. doi: 10.1371/journal.pone.0195292 (PMC5901783; doi:10.1371/journal.pone.0195292)
Supplement: S3 Table — (DOCX) [file pone.0195292.s003.docx]

**S3 Table. SMI selected 18-month performance indicators and requirements by country**

| Indicator # | Indicator description | Indicator requirements | BLZ | SLV | GTM | HND | CH (MEX) | NIC | PAN |
| --- | --- | --- | --- | --- | --- | --- | --- | --- | --- |
| 7000 | Health facilities with cold chain managed according to standards | Total procedural requirements per operating refrigerator | n/a | n/a | n/a | n/a | 4 | 2 | n/a |
| 7010/ 7060* | Health facilities with permanent availability of supplies and equipment necessary for pediatric, vaccination, and nutrition care | Number of equipment/medical forms | 12 | 3 | 4 | 11 | 8 | 6 | 0 |
|  |  | Number of vaccines | 5 | 0 | 6 | 0 | 5 | 6 | 0 |
|  |  | Number of pharmaceuticals | 7 | 3 | 6 | 5 | 5 | 4 | 5 |
|  |  | Total requirements | 24 | 6 | 16 | 16 | 18 | 16 | 5 |
| 7020 | Health facilities with permanent availability of supplies and equipment necessary for prenatal and postpartum care | Number of equipment/medical forms | 16 | 5 | 14 | n/a | 15 | 16 | 9 |
|  |  | Number of vaccines | 1 | 0 | 1 | n/a | 1 | 0 | 0 |
|  |  | Number of pharmaceuticals/lab reagents | 12 | 0 | 3 | n/a | 7 | 0 | 3 |
|  |  | Total requirements | 29 | 5 | 18 | n/a | 23 | 16 | 12 |
| 7030 | Health facilities that have the necessary supplies and equipment for providing emergency obstetric and neonatal care according to the norms | Number of equipment/medical forms | 12 | n/a | 12 | 0 | 12 | 0 | n/a |
|  |  | Number of pharmaceuticals | 14 | n/a | 13 | 4 | 13 | 6 | n/a |
|  |  | Total requirements | 26 | n/a | 25 | 4 | 25 | 6 | n/a |
| 7050 | Health facilities with permanent availability of modern family planning supplies (oral, injectable, barrier, IUD), according to the norms | Number of equipment | 0 | 0 | 0 | 0 | 1 | 0 | 1 |
|  |  | Number of family planning methods | 4 | 4 | 4 | 4 | 4 | 4 | 4 |
|  |  | Number of trained personnel | 2 | 0 | 0 | 0 | 2 | 4 | 0 |
|  |  | Total requirements | 6 | 4 | 4 | 4 | 7 | 8 | 5 |

*Indicator 7060 definition is health facilities with permanent availability of supplies and equipment for the childcare treatment of pneumonia and diarrhea. This indicator is only applicable for Honduras.
