# Supplementary material for: Supply-side interventions to improve health: Findings from the Salud Mesoamérica Initiative
Source: PLoS One. 2018 Apr 16;13(4):e0195292. doi: 10.1371/journal.pone.0195292 (PMC5901783; doi:10.1371/journal.pone.0195292)
Supplement: S1 Appendix — (DOCX) [file pone.0195292.s004.docx]

**S1 Appendix. Improvements in supplies and equipment from baseline to 18-month follow-up, by country**

Table 1A. Health facilities with availability of selected inputs and equipment necessary for basic child care, at the baseline, by country

|  | **Belize**  **% (95% CI)** | **El Salvador**  **% (95% CI)** | **Guatemala**  **% (95% CI)** | **Chiapas (MEX)**  **% (95% CI)** | **Nicaragua**  **% (95% CI)** | **Panama**  **% (95% CI)** |
| --- | --- | --- | --- | --- | --- | --- |
| **Equipment and medical forms** |  |  |  |  |  |  |
| Pediatric scale | 62·2 (44·8 - 77·5) |  | 87·3 (76·5 - 94·4) | 70·9 (57·1 - 82·4) | 81·1 (64·8 - 92·0) | 88·2 (72·5 - 96·7) |
| Pediatric stethoscope^1^ | 13·5 (4·5 - 28·8) |  | 17·6 (3·8 - 43·4) | 26 (14·6 - 40·3) | 8·1 (1·7 - 21·9) | 19 (5·4 - 41·9) |
| Neonatal/pediatric blood pressure apparatus^2^ | 25 (0·6 - 80·6) |  | 12·5 (1·6 - 38·3) | 22·2 (6·4 - 47·6) |  | 23·1 (5·0 - 53·8) |
| Thermometer^3^ | 60 (40·6 - 77·3) |  | 87·2 (74·3 - 95·2) | 97·3 (85·8 - 99·9) | 18·8 (7·2 - 36·4) | 68·4 (43·4 - 87·4) |
| Growth & development card | 100 (90·5 - 100) |  | 98·4 (91·5 - 100) | 89·1 (77·8 - 95·9) | 86·5 (71·2 - 95·5) | 88·2 (72·5 - 96·7) |
| **Pharmacy inputs** |  |  |  |  |  |  |
| Packets/envelopes of oral rehydration salts | 52 (31·3 - 72·2) | 89·7 (78·8 - 96·1) | 85·7 (74·6 - 93·3) | 75 (61·1 - 86·0) | 89·2 (74·6 - 97·0) | 88·2 (72·5 - 96·7) |
| Albendazole/mebendazole^4^ | 64 (42·5 - 82·0) | 48·3 (35·0 - 61·8) | 63·5 (50·4 - 75·3) | 76·9 (63·2 - 87·5) | 94·6 (81·8 - 99·3) | 85·3 (68·9 - 95·0) |
| Ferrous sulfate/micronutrients^4^ | 20 (6·8 - 40·7) | 32 (14·9 - 53.5) | 88·9 (78·4 - 95·4) | 57·7 (43·2 – 71·3) | 97·3 (85·8 - 99·9) | 88·2 (72·5 - 96·7) |
| Antibiotics^5^ | 44 (24·4 - 65·1) |  | 78·6 (49·2 - 95·3) | 54·2 (32·8 - 74·4) | 100 (59·0 - 100) | 94·1 (71·3 - 99·9) |
| **Vaccines^6^** |  |  |  |  |  |  |
| Pentavalent/(HepB + DPT) | 100 (69·2 - 100) | 95·2 (76·2 - 99·9) | 94·3 (80·8 - 99·3) | 74·1 (53·7 - 88·9) | 95·2 (76·2 - 99·9) | 100 (76·8 - 100) |
| Polio | 100 (69·2 - 100) | 81·0 (58·1 - 94·6) | 91·4 (76·9 - 98·2) | 40·7 (22·4 - 61·2) | 95·2 (76·2 - 99·9) | 92·9 (66·1 - 99·8) |
| Rotavirus |  | 71·4 (47·8 - 88·7) | 91·4 (76·9 - 98·2) | 74·1 (53·7 - 88·9) | 85·7 (63·7 - 97·0) | 100 (76·8 - 100) |
| Pneumococcal conjugate |  | 71·4 (47·8 - 88·7) | 91·4 (76·9 - 98·2) | 37 (19·4 - 57·6) | 61·9 (38·4 - 81·9) | 100 (76·8 - 100) |
| MMR | 100 (69·2 - 100) | 78·9 (54·4 - 93·9) | 94·3 (80·8 - 99·3) | 81·5 (61·9 - 93·7) | 95·2 (76·2 - 99·9) | 92·9 (66·1 - 99·8) |
| BCG | 100 (69·2 - 100) | 76·2 (52·8 - 91·8) | 97·1 (85·1 - 99·9) | 70·4 (49·8 - 86·2) | 28·6 (11·3 - 52·2) | 100 (76·8 - 100) |
| **Cold chain standards** |  |  |  |  |  |  |
| Monitoring charts observed for each fridge |  |  |  | 70·8 (48·9 - 87·4) | 57·1 (37·2 - 75·5) |  |
| Temp. 2-8°C on the day of the survey |  |  |  | 70·8 (48·9 - 87·4) |  |  |
| Temp. recorded twice daily during last 30 days |  |  |  | 70·8 (48·9 - 87·4) | 28·6 (13·2 - 48·7) |  |
| Temp. 2-8°C for last 30 days |  |  |  | 95·8 (78·9 - 99·9) |  |  |
| ^1^Data missing for 4 ambulatory facilities with a doctor in Guatemala; not measured in ambulatory facilities without a doctor in Guatemala, Mexico, & Panama; not measured in complete facilities in Nicaragua; ^2^Only applicable at basic and complete facilities; measured only in complete facilities in Nicaragua; ^3^Only applicable at ambulatory facilities; data missing from 3 ambulatory facilities in Belize; ^4^El Salvador only measured albendazole & micronutrients; micronutrients only measured in El Salvador if facility stores vaccines; ^5^Antibiotics = ampicillin/penicillin (benzathine/crystalline)/amoxicillin; ^6^Applicable only if facility stores vaccines and provides basic child care | | | | | | |

Table 1B. Health facilities with availability of selected inputs and equipment necessary for basic child care, at the follow-up, by country

|  | **Belize**  **% (95% CI)** | **El Salvador**  **% (95% CI)** | **Guatemala**  **% (95% CI)** | **Chiapas (MEX)**  **% (95% CI)** | **Nicaragua**  **% (95% CI)** | **Panama**  **% (95% CI)** |
| --- | --- | --- | --- | --- | --- | --- |
| **Equipment and medical forms** |  |  |  |  |  |  |
| Pediatric scale/salter scale^1^ | 100 (90·5 - 100) | 96·1 (86·5 - 99·5) | 100 (66·4 - 100) | 81·7 (69·6 - 90·5) | 100 (93·3 - 100) | 78·9 (62·7 - 90·4) |
| Pediatric stethoscope^2^ | 13·5 (4·5 - 28·8) | 86·3 (73·7 - 94·3) | 67·3 (53·3 - 79·3) | 26·7 (16·1 - 39·7) | 94·4 (72·7 - 99·9) | 28·9 (15·4 - 45·9) |
| Neonatal/pediatric blood pressure apparatus^3^ | 100 (39·8 - 100) |  | 44·4 (21·5 - 69·2) | 50 (23·0 - 77·0) | 90·9 (58·7 - 99·8) | 35·3 (14·2 - 61·7) |
| Thermometer (ambulatory facilities) | 93·9 (79·8 - 99·3) | 100 (93·0 - 100) | 94·6 (81·8 - 99·3) | 95·6 (84·9 - 99·5) | 95·2 (83·8 - 99·4) | 85·7 (63·7 - 97·0) |
| Growth & development card | 100 (90·5 - 100) |  |  | 90 (79·5 - 96·2) | 92·5 (81·8 - 97·9) | 76·3 (59·8 - 88·6) |
| **Pharmacy inputs** |  |  |  |  |  |  |
| Packets/envelopes of oral rehydration salts | 94·6 (81·8 - 99·3) | 98 (89·6 - 100) | 92·7 (82·4 - 98·0) | 100 (94·0 - 100) | 100 (93·3 - 100) | 71·1 (54·1 - 84·6) |
| Albendazole/mebendazole | 94·6 (81·8 - 99·3) | 100 (93·0 - 100) | 94·5 (84·9 - 98·9) | 100 (94·0 - 100) | 94·3 (84·3 - 98·8) | 78·9 (62·7 - 90·4) |
| Ferrous sulfate/micronutrients | 10·8 (3·0 - 25·4) | 100 (93·0 - 100) | 94·5 (84·9 - 98·9) | 90 (79·5 - 96·2) | 100 (93·3 - 100) | 78·9 (62·7 - 90·4) |
| Antibiotics^3^ | 40·5 (24·8 - 57·9) | 100 (93·0 - 100) | 100 (83·2 - 100) | 98 (89·6 - 100) | 100 (93·3 - 100) | 100 (83·2 - 100) |
| **Vaccines** |  |  |  |  |  |  |
| Pentavalent/(HepB + DPT) | 100 (78·2 - 100) | 100 (90·7 - 100) | 68·2 (52·4 - 81·4) | 57·1 (37·2 - 75·5) | 100 (88·4 - 100) | 90·9 (70·8 - 98·9) |
| Polio | 100 (78·2 - 100) | 97·4 (86·2 - 99·9) | 52·3 (36·7 - 67·5) | 46·4 (27·5 - 66·1) | 100 (88·4 - 100) | 81·8 (59·7 - 94·8) |
| Rotavirus |  | 97·4 (86·2 - 99·9) | 52·3 (36·7 - 67·5) | 78·6 (59·0 - 91·7) | 100 (88·4 - 100) | 86·4 (65·1 - 97·1) |
| Pneumococcal conjugate |  | 94·7 (82·3 - 99·4) | 31·8 (18·6 - 47·6) | 42·9 (24·5 - 62·8) | 96·7 (82·8 - 99·9) | 77·3 (54·6 - 92·2) |
| MMR | 100 (78·2 - 100) | 97·4 (86·2 - 99·9) | 43·2 (28·3 - 59·0) | 85·7 (67·3 - 96·0) | 100 (88·4 - 100) | 72·7 (49·8 - 89·3) |
| BCG | 100 (78·2 - 100) | 92·1 (78·6 - 98·3) | 45·5 (30·4 - 61·2) | 50 (30·6 - 69·4) | 100 (88·4 - 100) | 81·8 (59·7 - 94·8) |
| **Cold chain standards** |  |  |  |  |  |  |
| Monitoring charts observed for each fridge |  |  |  | 100 (87·2 - 100) | 92·6 (75·7 - 99·1) |  |
| Temp. 2-8°C on the day of the survey |  |  |  | 92·6 (75·7 - 99·1) |  |  |
| Temp. recorded twice daily during the last 30 days^4^ |  |  |  | 85·2 (66·3 - 95·8) | 88·9 (70·8 - 97·6) |  |
| Temperature 2-8°C for the last 30 days |  |  |  | 100 (87·2 - 100) |  |  |
| ^1^Missing data from 8 facilities in Guatemala; ^2^Not measured in health posts in Nicaragua; ^3^Antibiotics = ampicillin/penicillin (benzathine/crystalline)/amoxicillin; ^4^Nicaragua specified to exclude weekends and local holidays | | | | | | |
|  | | | | | | |

Table 2A. Health facilities with availability of selected inputs and equipment necessary for antenatal and postpartum care, at the baseline, by country

|  | **Belize**  **% (95% CI)** | **El Salvador**  **% (95% CI)** | **Guatemala**  **% (95% CI)** | **Chiapas (MEX)**  **% (95% CI)** | **Nicaragua**  **% (95% CI)** | **Panama**  **% (95% CI)** |
| --- | --- | --- | --- | --- | --- | --- |
| **Equipment and medical forms** |  |  |  |  |  |  |
| Standing scale (with or without height rod) | 60 (42·1 - 76·1) | 98·3 (90·8 - 100) | 70·3 (57·6 - 81·1) | 70·9 (57·1 - 82·4) | 89·2 (74·6 - 97·0) | 79·2 (57·8 - 92·9) |
| Height rod^2^ | 60 (42·1 - 76·1) |  | 82·8 (71·3 - 91·1) | 72·7 (59·0 - 83·9) | 51·4 (34·4 - 68·1) | 41·7 (22·1 - 63·4) |
| CLAP obstetrical tape | 54·3 (36·6 - 71·2) |  | 73·4 (60·9 - 83·7) | 29·1 (17·6 - 42·9) | 75·7 (58·8 - 88·2) | 45·8 (25·6 - 67·2) |
| Gooseneck lamp | 48·6 (31·4 - 66·0) | 56·9 (43·2 - 69·8) | 57·8 (44·8 - 70·1) | 67·3 (53·3 - 79·3) | 54·1 (36·9 - 70·5) | 25 (9·8 - 46·7) |
| Blood pressure apparatus | 48·6 (31·4 - 66·0) | 89·7 (78·8 - 96·1) | 81·3 (69·5 - 89·9) | 85·5 (73·3 - 93·5) | 89·2 (74·6 - 97·0) | 83·3 (62·6 - 95·3) |
| Stethoscope | 57·1 (39·4 - 73·7) | 91·4 (81·0 - 97·1) | 84·4 (73·1 - 92·2) | 85·5 (73·3 - 93·5) | 86·5 (71·2 - 95·5) | 91·7 (73·0 - 99·0) |
| Perinatal maternal medical history | 60 (42·1 - 76·1) |  | 93·8 (84·8 - 98·3) | 90·9 (80·0 - 97·0) | 83·8 (68·0 - 93·8) | 83·3 (65·3 - 94·4) |
| Perinatal maternal card | 60 (42·1 - 76·1) |  | 73·4 (60·9 - 83·7) | 90·9 (80·0 - 97·0) | 73 (55·9 - 86·2) | 90 (73·5 - 97·9) |
| Gynecological exam table/stretcher^1^ | 62·5 (43·7 - 78·9) |  | 83·3 (70·7 - 92·1) | 82 (68·6 - 91·4) | 91·9 (78·1 - 98·3) | 58·3 (36·6 - 77·9) |
| **Pharmacy inputs** |  |  |  |  |  |  |
| (Iron + folic acid)/multivitamins | 65·2 (42·7 - 83·6) |  | 85·9 (75·0 - 93·4) | 71·7 (57·7 - 83·2) | 73·0 (55·9 - 86·2) | 83·3 (65·3 - 94·4) |
| Cephalexin (basic & complete facilities) | 75 (19·4 - 99·4) |  | 11·8 (1·5 - 36·4) | 31·3 (11·0 - 58·7) | 0 (0·0 - 97·5) | 30·8 (9·1 - 61·4) |
| Tetanus vaccine (if facility stores vaccines) | 70 (34·8 - 93·3) | 81 (58·1 - 94·6) | 93·3 (81·7 - 98·6) | 28·6 (13·2 - 48·7) | 32·1 (15·9 - 52·4) | 82·4 (56·6 - 96·2) |
| **Laboratory inputs^2^** |  |  |  |  |  |  |
| Rapid syphilis test/dark field microscope/enzyme immunoassay equipment | 75 (19·4 - 99·4) |  | 57·1 (18·4 - 90·1) | 60 (32·3 - 83·7) | 60 (14·7 - 94·7) | 100 (39·8 - 100) |
| Rapid HIV test/fluorescence microscope | 50 (6·8 - 93·2) |  | 71·4 (29·0 - 96·3) | 53·3 (26·6 - 78·7) | 60 (14·7 - 94·7) | 100 (39·8 - 100) |
| Urine protein strips/urinalysis | 100 (39·8 - 100) |  | 57·1 (18·4 - 90·1) | 73·3 (44·9 - 92·2) | 100 (47·8 - 100) | 100 (39·8 - 100) |
| Blood glucose meter (complete facilities) | 0 (0·0 - 84·2) |  | 66·7 (9·4 - 99·2) | 50 (11·8 - 88·2) |  |  |
| HemoCue/automated cell counter | 50 (6·8 - 93·2) |  | 42·9 (9·9 - 81·6) | 66·7 (38·4 - 88·2) | 100 (47·8 - 100) | 75 (19·4 - 99·4) |
| Pregnancy test (basic facilities) | 100 (15·8 - 100) |  | 75 (19·4 - 99·4) | 100 (66·4 - 100) | 100 (47·8 - 100) | 100 (39·8 - 100) |
| ^1^Not applicable for mobile units; ^2^Only applicable to basic/complete facilities with a laboratory; not all alternatives listed in this table were represented in all countries at all facility levels | | | | | | |

Table 2B. Health facilities with availability of selected inputs and equipment necessary for antenatal and postpartum care, at the follow-up, by country

|  | **Belize**  **% (95% CI)** | **El Salvador**  **% (95% CI)** | **Guatemala**  **% (95% CI)** | **Chiapas (MEX)**  **% (95% CI)** | **Nicaragua**  **% (95% CI)** | **Panama**  **% (95% CI)** |
| --- | --- | --- | --- | --- | --- | --- |
| **Equipment and medical forms** |  |  |  |  |  |  |
| Standing scale (with or without height rod) | 79·3 (60·3 - 92·0) | 100 (93·0 - 100) | 100 (93·7 - 100) | 98·3 (91·1 - 100) | 98·2 (90·4 - 100) | 87·5 (71·0 - 96·5) |
| Height rod^2^ | 100 (87·7 - 100) | 100 (93·0 - 100) | 98·2 (90·6 - 100) | 100 (94·0 - 100) | 100 (93·6 - 100) | 100 (89·1 - 100) |
| CLAP obstetrical tape | 96·6 (82·2 - 99·9) | 98 (89·6 - 100) | 91·2 (80·7 - 97·1) | 100 (94·0 - 100) | 100 (93·6 - 100) | 87·5 (71·0 - 96·5) |
| Gooseneck lamp | 96·6 (82·2 - 99·9) | 100 (93·0 - 100) | 77·2 (64·2 - 87·3) | 95 (86·1 - 99·0) | 83·9 (71·7 - 92.4) | 71·9 (53·3 - 86·3) |
| Blood pressure apparatus | 100 (88·1 - 100) | 100 (93·0 - 100) | 100 (93·7 - 100) | 95 (86·1 - 99·0) | 98·2 (90·4 - 100) | 93·8 (79·2 - 99·2) |
| Stethoscope | 100 (88·1 - 100) | 100 (93·0 - 100) | 96·5 (87·9 - 99·6) | 100 (94·0 - 100) | 98·2 (90·4 - 100) | 93·8 (79·2 - 99·2) |
| Perinatal maternal medical history | 96·6 (82·2 - 99·9) | 100 (93·0 - 100) | 98·2 (90·6 - 100) | 91·7 (81·6 - 97·2) | 100 (93·6 - 100) | 90·6 (75·0 - 98·0) |
| Perinatal maternal card | 96·6 (82·2 - 99·9) | 100 (93·0 - 100) | 86 (74·2 - 93·7) | 88·3 (77·4 - 95·2) | 100 (93·6 - 100) | 90·6 (75·0 - 98·0) |
| Gynecological exam table/stretcher^1^ | 100 (88·1 - 100) | 100 (93·0 - 100) | 72·7 (59·0 - 83·9) | 100 (93·0 - 100) | 98·2 (90·4 - 100) | 90 (73·5 - 97·9) |
| **Pharmacy inputs** |  |  |  |  |  |  |
| (Iron + folic acid)/multivitamins | 93·3 (77·9 - 99·2) | 96·1 (86·5 - 99·5) | 94·6 (85·1 - 98·9) | 98·3 (91·1 - 100) | 91·1 (80·4 - 97·0) | 87·5 (71·0 - 96·5) |
| Cephalexin (basic & complete facilities) | 100 (39·8 - 100) |  | 0 (0.0 - 18·5) | 100 (76·8 - 100) | 90·9 (58·7 - 99·8) | 100 (80·5 - 100) |
| Tetanus vaccine (if facility stores vaccines) | 100 (78·2 - 100) |  | 81·3 (67·4 - 91·1) | 85·7 (67·3 - 96·0) | 96·9 (83·8 - 99·9) | 81·8 (59·7 - 94·8) |
| **Laboratory inputs^2^** |  |  |  |  |  |  |
| Rapid syphilis test/dark field microscope/enzyme immunoassay equipment | 100 (29·2 - 100) |  | 78·6 (49·2 - 95·3) | 85·7 (57·2 - 98·2) | 90·9 (58·7 - 99·8) | 100 (63·1 - 100) |
| Rapid HIV test/fluorescence microscope | 100 (29·2 - 100) |  | 92·9 (66·1 - 99·8) | 100 (76·8 - 100) | 100 (71·5 - 100) | 100 (63·1 - 100) |
| Urine protein strips/urinalysis | 100 (29·2 - 100) |  | 92·9 (66·1 - 99·8) | 92·9 (66·1 - 99·8) | 100 (71·5 - 100) | 87·5 (47·3 - 99·7) |
| Blood glucose meter (complete facilities) | 0 (0·0 - 84·2) |  | 75 (19·4 - 99·4) | 28·6 (3·7 - 71·0) |  |  |
| HemoCue/automated cell counter | 100 (29·2 - 100) |  | 92·9 (66·1 - 99·8) | 78·6 (49·2 - 95·3) | 100 (71·5 - 100) | 100 (63·1 - 100) |
| Pregnancy test (basic facilities) | 100 (2·5 - 100) |  | 50 (18·7 - 81·3) | 100 (59·0 - 100) | 100 (71·5 - 100) | 100 (63·1 - 100) |
| ^1^Not applicable for mobile units; ^2^Only applicable to basic/complete facilities with a laboratory; not all alternatives listed in this table were represented in all countries at all facility levels | | | | | | |

Table 3A. Health facilities with availability of selected inputs necessary for delivery and emergency obstetric care, at the baseline, by country

|  | **Belize**  **% (95% CI)** | **Guatemala**  **% (95% CI)** | **Honduras**  **% (95% CI)** | **Chiapas (MEX)**  **% (95% CI)** | **Nicaragua**  **% (95% CI)** |
| --- | --- | --- | --- | --- | --- |
| **Equipment^1^** |  |  |  |  |  |
| Pinard stethoscope/portable Doppler | 75 (19·4 - 99·4) | 88·2 (63·6 - 98·5) | 75 (34·9 - 96·8) | 61·5 (31·6 - 86·1) | 40 (5·3 - 85·3) |
| Autoclave/dry heat sterilizer | 25 (0·6 - 80·6) | 70·6 (44·0 - 89·7) | 87·5 (47·3 - 99·7) | 46·2 (19·2 - 74·9) | 20 (0·5 - 71·6) |
| Oxygen tank | 100 (39·8 - 100) | 64·7 (38·3 - 85·8) | 100 (63·1 - 100) | 61·5 (31·6 - 86·1) | 60 (14·7 - 94·7) |
| Adult resuscitation bag | 100 (39·8 - 100) | 64·7 (38·3 - 85·8) | 87·5 (47·3 - 99·7) | 53·8 (25·1 - 80·8) | 40 (5·3 - 85·3) |
| Neonatal resuscitation bag | 100 (39·8 - 100) | 52·9 (27·8 - 77·0) | 100 (63·1 - 100) | 69·2 (38·6 - 90·9) | 40 (5·3 - 85·3) |
| Laryngoscope | 25 (0·6 - 80·6) | 29·4 (10·3 - 56·0) | 75 (34·9 - 96·8) | 61·5 (31·6 - 86·1) | 40 (5·3 - 85·3) |
| MVA kit | 0 (0·0 - 60·2) | 23·5 (6·8 - 49·9) | 0 (0·0 - 36·9) | 30·8 (9·1 - 61·4) | 100 (47·8 - 100) |
| **Pharmacy inputs^1^** |  |  |  |  |  |
| Oxytocin/ergonovine/ergometrine | 100 (39·8 - 100) | 88·2 (63·6 - 98·5) | 100 (63·1 - 100) | 58·3 (27·7 - 84·8) | 100 (47·8 - 100) |
| Dexamethasone/betamethasone | 100 (39·8 - 100) | 82·4 (56·6 - 96·2) | 62·5 (24·5 - 91·5) | 25 (5·5 - 57·2) | 100 (47·8 - 100) |
| Magnesium sulfate | 100 (39·8 - 100) | 88·2 (63·6 - 98·5) | 100 (63·1 - 100) | 41·7 (15·2 - 72·3) | 80 (28·4 - 99·5) |
| Hydralazine/hydralazine chlorhydrate | 75 (19·4 - 99·4) | 35·3 (14·2 - 61·7) | 75 (34·9 - 96·8) | 33·3 (9·9 - 65·1) | 80 (28·4 - 99·5) |
| ^1^Not all alternatives listed in this table were represented in all countries at all facility levels; ^2^Measured only at basic facilities in Belize | | | | | |

Table 3B. Health facilities with availability of selected inputs necessary for delivery and emergency obstetric care, at the follow-up, by country

|  | **Belize**  **% (95% CI)** | **Guatemala**  **% (95% CI)** | **Honduras**  **% (95% CI)** | **Chiapas (MEX)**  **% (95% CI)** | **Nicaragua**  **% (95% CI)** |
| --- | --- | --- | --- | --- | --- |
| **Equipment^1^** |  |  |  |  |  |
| Pinard stethoscope/portable Doppler | 50 (6·8 - 93·2) | 84·6 (54·6 - 98·1) | 85·7 (42·1 - 99·6) | 85·7 (57·2 - 98·2) | 100 (71·5 - 100) |
| Autoclave/dry heat sterilizer | 100 (39·8 - 100) | 91·7 (61·5 - 99·8) | 100 (59·0 - 100) | 85·7 (57·2 - 98·2) | 90·9 (58·7 - 99·8) |
| Oxygen tank | 100 (39·8 - 100) | 92·3 (64·0 - 99·8) | 100 (59·0 - 100) | 92·9 (66·1 - 99·8) | 90·9 (58·7 - 99·8) |
| Adult resuscitation bag | 100 (39·8 - 100) | 100 (75·3 - 100) | 100 (59·0 - 100) | 78·6 (49·2 - 95·3) | 90·9 (58·7 - 99·8) |
| Neonatal resuscitation bag | 75 (19·4 - 99·4) | 92·3 (64·0 - 99·8) | 100 (59·0 - 100) | 78·6 (49·2 - 95·3) | 100 (71·5 - 100) |
| Laryngoscope | 100 (39·8 - 100.0) | 69·2 (38·6 - 90·9) | 100 (59·0 - 100) | 85·7 (57·2 - 98·2) | 90·9 (58·7 - 99·8) |
| MVA kit^2^ | 50 (6·8 - 93·2) | 53·8 (25·1 - 80·8) |  | 42·9 (17·7 - 71·1) | 100 (71·5 - 100) |
| **Pharmacy inputs^1^** |  |  |  |  |  |
| Oxytocin/ergonovine/ergometrine | 100 (39·8 - 100) | 100 (73·5 - 100) | 100 (59·0 - 100) | 100 (76·8 - 100) | 100 (71·5 - 100) |
| Dexamethasone/betamethasone | 100 (39·8 - 100) | 83·3 (51·6 - 97·9) | 100 (59·0 - 100) | 100 (76·8 - 100) | 100 (71·5 - 100) |
| Magnesium sulfate | 100 (39·8 - 100) | 100 (73·5 - 100) | 100 (59·0 - 100) | 85·7 (57·2 - 98·2) | 100 (71·5 - 100) |
| Hydralazine ampoule | 100 (39·8 - 100) | 83·3 (51·6 - 97·9) | 100 (59·0 - 100) | 100 (76·8 - 100) | 100 (71·5 - 100) |
| ^1^Not all alternatives listed in this table were represented in all countries at all facility levels; ^2^Measured only at complete facilities in Honduras | | | | | |

Table 4A. Health facilities with availability of selected modern family planning supplies, at the baseline, by country

|  | **Belize**  **% (95% CI)** | **El Salvador**  **% (95% CI)** | **Guatemala**  **% (95% CI)** | **Honduras**  **% (95% CI)** | **Chiapas (MEX)**  **% (95% CI)** | **Nicaragua**  **% (95% CI)** | **Panama**  **% (95% CI)** |
| --- | --- | --- | --- | --- | --- | --- | --- |
| **FP method** |  |  |  |  |  |  |  |
| Male condom | 89·5 (66·9 - 98·7) | 89·7 (78·8 - 96·1) | 83·9 (72·3 - 92·0) | 94·9 (85·9 - 98·9) | 93·9 (83·1 - 98·7) | 78·4 (61·8 - 90·2) | 100 (76·8 - 100) |
| Any pill | 100 (82·4 - 100) | 87·9 (76·7 - 95·0) | 75·8 (63·3 - 85·8) | 98·3 (90·9 - 100) | 89·8 (77·8 - 96·6) | 78·4 (61·8 - 90·2) | 92·9 (66·1 - 99·8) |
| Any injectable | 78·9 (54·4 - 93·9) | 89·7 (78·8 - 96·1) | 98·4 (91·3 - 100) | 96·6 (88·3 - 99·6) | 89·8 (77·8 - 96·6) | 97·3 (85·8 - 99·9) | 85·7 (57·2 - 98·2) |
| IUD^1^ | 100 (39·8 - 100) |  | 70·6 (44·0 - 89·7) | 92·9 (66·1 - 99·8) | 71·4 (41·9 - 91·6) | 80 (28·4 - 99·5) | 42·9 (17·7 - 71·1) |
| IUD insertion kit^1^ | 25 (0·6 - 80·6) |  | 76·5 (50·1 - 93·2) | 100 (76·8 - 100) | 71·4 (41·9 - 91·6) |  | 14·3 (1·8 - 42·8) |
| ^1^ Only applicable at basic and complete facilities | | |  |  |  |  |  |

Table 4B. Health facilities with availability of selected modern family planning supplies, at the follow-up, by country

|  | **Belize**  **% (95% CI)** | **El Salvador**  **% (95% CI)** | **Guatemala**  **% (95% CI)** | **Honduras**  **% (95% CI)** | **Chiapas (MEX)**  **% (95% CI)** | **Nicaragua**  **% (95% CI)** | **Panama**  **% (95% CI)** |
| --- | --- | --- | --- | --- | --- | --- | --- |
| **FP Method** |  |  |  |  |  |  |  |
| Male condom | 100 (83·2 - 100) | 100 (93·0 - 100) | 94·6 (85·1 - 98·9) | 100 (93·7 - 100) | 100 (93·9 - 100) | 98·2 (90·4 - 100) | 100 (85·8 - 100) |
| Any pill | 100 (83·2 - 100) | 100 (93·0 - 100) | 91·1 (80·4 - 97·0) | 96·5 (87·9 - 99·6) | 88·1 (77·1 - 95·1) | 98·2 (90·4 - 100) | 100 (85·8 - 100) |
| Any injectable | 95 (75·1 - 99·9) | 98 (89·6 - 100) | 98·2 (90·4 - 100) | 100 (93·7 - 100) | 100 (93·9 - 100) | 100 (93·6 - 100) | 100 (85·8 - 100) |
| IUD | 100 (39·8 - 100) |  | 94·7 (74·0 - 99·9) | 100 (71·5 - 100) | 100 (75·3 - 100) | 100 (71·5 - 100) | 100 (80·5 - 100) |
| IUD insertion kit | 100 (39·8 - 100) |  | 89·5 (66·9 - 98·7) | 83·3 (51·6 - 97·9) | 84·6 (54·6 - 98·1) | 100 (71·5 - 100) | 94·1 (71·3 - 99·9) |
| 1 Only applicable at basic and complete facilities; missing data from one complete facility in Honduras | | | | | | | |
